# The effect of nebulized N-acetylcysteine on the phlegm of chronic obstructive pulmonary disease: the NEWEST study

**DOI:** 10.1186/s12890-024-03243-y

**Published:** 2024-09-02

**Authors:** Chin Kook Rhee, Seong Yong Lim, Won-Yeon Lee, Ji Ye Jung, Yong Bum Park, Chang Youl Lee, Yong Il Hwang, Jin Woo Song, Won-Il Choi, Kwang Ha Yoo, Ki Uk Kim, Yu-Il Kim, Tae-Hyung Kim, Seong Ju Park, Kyeong-Cheol Shin, Soo-Jung Um, Hyoung Kyu Yoon, Ho Sung Lee, Deog Kyeom Kim, Ah Young Leem, on Behalf of the Korean Pulmonary Rehabilitation Study Group

**Affiliations:** 1 Division of Pulmonary and Critical Care Medicine, Department of Internal Medicine, Seoul St. Mary’s Hospital, College of Medicine, The Catholic University of Korea, Seoul, South Korea; 2 Division of Pulmonary and Critical Care Medicine, Department of Medicine, Kangbuk Samsung Hospital, Sungkyunkwan University School of Medicine, Seoul, South Korea; 3 Division of Pulmonary, Allergy, and Critical Care Medicine, Department of Internal Medicine, Yonsei University Wonju Severance Christian Hospital, Yonsei University Wonju College of Medicine, Wonju, South Korea; 4 Division of Pulmonary and Critical Care Medicine, Department of Internal Medicine, Severance Hospital, Yonsei University College of Medicine, Seoul, South Korea; 5 Division of Pulmonary, Allergy and Critical Care Medicine, Department of Internal Medicine, Hallym University Kangdong Sacred Heart Hospital, Seoul, South Korea; 6 Division of Pulmonary, Allergy and Critical Care Medicine, Department of Internal Medicine, Chuncheon Sacred Heart Hospital, Hallym University College of Medicine, Chuncheon, South Korea; 7 Division of Pulmonary, Allergy and Critical Care Medicine, Department of Internal Medicine, Hallym University Medical Center, Hallym University College of Medicine, Anyang, South Korea; 8 Division of Pulmonary and Critical Care Medicine, Asan Medical Center, University of Ulsan College of Medicine, Seoul, South Korea; 9 Department of Internal Medicine, Myongji Hospital, Hanyang University College of Medicine, Goyang, South Korea; 10 Division of Pulmonary, Allergy and Critical Care Medicine, Department of Internal Medicine, Konkuk University School of Medicine, 120 Neungdong-Ro, Gwangjin-Gu, Seoul, 05029 South Korea; 11 Division of Pulmonary, Allergy and Critical Care Medicine, Department of Internal Medicine, Pusan National University School of Medicine, Busan, South Korea; 12 Division of Pulmonary Medicine, Department of Internal Medicine, Chonnam National University Hospital, Gwangju, South Korea; 13 Division of Pulmonary and Critical Care Medicine. Department of Internal Medicine, Hanyang University Guri Hospital, Hanyang University College of Medicine, Guri, South Korea; 14 Division of Pulmonary, Allergy, and Critical Care Medicine, Department of Internal Medicine, Jeonbuk National University Medical School, Jeonju, South Korea; 15 Division of Pulmonology and Allergy, Department of Internal Medicine, Regional Center for Respiratory Disease, College of Medicine, Yeungnam University, Daegu, South Korea; 16 Division of Respiratory Medicine, Department of Internal Medicine, Dong-A University Medical Center, Dong-A University College of Medicine, Busan, South Korea; 17 Division of Pulmonary and Critical Care Medicine, Department of Internal Medicine, Yeouido St. Mary’s Hospital, College of Medicine, The Catholic University of Korea, Seoul, South Korea; 18 Division of Respiratory Medicine, Soon Chun Hyang University Hospital Cheonan, Cheonan, South Korea; 19 Division of Pulmonary and Critical Care Medicine, Department of Internal Medicine, Seoul Metropolitan Government-Seoul National University Boramae Medical Center, Seoul National University College of Medicine, Seoul, South Korea; 20 The Korean Academy of Tuberculosis and Respiratory Diseases (KATRD), 101-605, 58, Banpo-Daero, Seocho-Gu, Seoul, 06652 South Korea

**Keywords:** N-acetylcysteine, Nebulizer, Chronic obstructive pulmonary disease, Chronic bronchitis

## Abstract

**Background:**

Phlegm is prevalent symptom in patients with chronic obstructive pulmonary disease (COPD). Few studies have investigated the effectiveness of N-acetylcysteine (NAC) nebulizer therapy in COPD patients. We evaluated the effect of nebulized NAC on the improvement of phlegm symptom in COPD patients.

**Methods:**

This was a 12-week, prospective, single-arm, open-label, phase IV multi-center trial (NCT05102305, Registration Date: 20-October-2021). We enrolled patients aged ≥ 40 years with post bronchodilator forced expiratory volume in one second/forced vital capacity (FEV_1_/FVC) < 0.7 and COPD assessment test (CAT) phlegm score ≥ 2; the patients were current or ex-smoker with smoking pack-years ≥ 10. The primary endpoint was to determine the change in CAT phlegm score at 12 weeks compared to the baseline. Patients were assessed at baseline, 4, 8, and 12 weeks of treatment using the CAT score.

**Results:**

In total, 100 COPD patients were enrolled from 10 hospitals. The mean age of the patients was 71.42 ± 8.20 years, with 19.78% being current-smokers and 80.22% being ex-smokers. The mean smoking pack-years was 40.32 ± 35.18. The mean FVC, FEV_1_, and FEV_1_/FVC were 3.94 L (75.44%), 2.22 L (58.50%), and 0.53, respectively. The CAT phlegm score at baseline was 3.47 ± 1.06, whereas after 12 weeks of nebulized NAC it significantly decreased to 2.62 ± 1.30 (*p* < 0.01). More than half (53.5%) of the patients expressed satisfaction with the effects of nebulized NAC therapy. Adverse events occurred in 8 (8.0%) patients. Notably, no serious adverse drug reactions were reported.

**Conclusion:**

In this study, we have established the effectiveness and safety of nebulized NAC over 12 weeks.

## Introduction

Chronic obstructive pulmonary disease (COPD), a leading cause of morbidity and mortality worldwide [1], is characterized by persistent respiratory symptoms, such as cough, sputum production, and dyspnea [2]. Phlegm, a common symptom in patients with COPD, contributes to mucus production [3]; its failure to be expectorated can lead to airflow limitation and exacerbate inflammatory small airway disease [4, 5]. Moreover, mucus hypersecretion plays a pivotal role in the pathophysiology of various severe respiratory conditions, including asthma, COPD, and cystic fibrosis [6]. The accumulation of mucus provides a breeding ground for chronic bacterial colonization and infection [3]. Therefore, alleviating phlegm symptoms and promoting expectoration in COPD patients can significantly improve the quality of life and prevent disease progression.

Alterations in the expression of mucin genes, production of the mucin, and dehydration of the mucus layer are main pathogenesis in patients with COPD. Pathogens, such as respiratory viruses and bacteria, may upregulate mucin-secreting cell differentiation and mucin secretion in COPD. Currently, our knowledge of the effects of the drugs already available on the market that target mucin is extremely limited. Thus, well-controlled clinical trials are needed [7].

N-acetylcysteine (NAC) is a commonly prescribed oral medication in COPD patients. Previous studies have demonstrated that NAC effectively reduces exacerbation in COPD patients [8]. Furthermore, recent study showed that intravenous administration of NAC significantly decreases sputum viscosity and expectoration difficulty in patients with abnormal mucus secretion [9]. However, few studies have investigated the effectiveness of nebulized NAC in COPD patients. There has been no long-term study to establish the effectiveness of nebulized NAC. Additionally, the sample size of previous studies on nebulized NAC was small. Moreover, there have been no multicenter prospective trials.

We evaluated the effect of nebulized NAC on the improvement of phlegm symptoms in COPD patients.

## Methods

### Study design

**Fig. 1:**
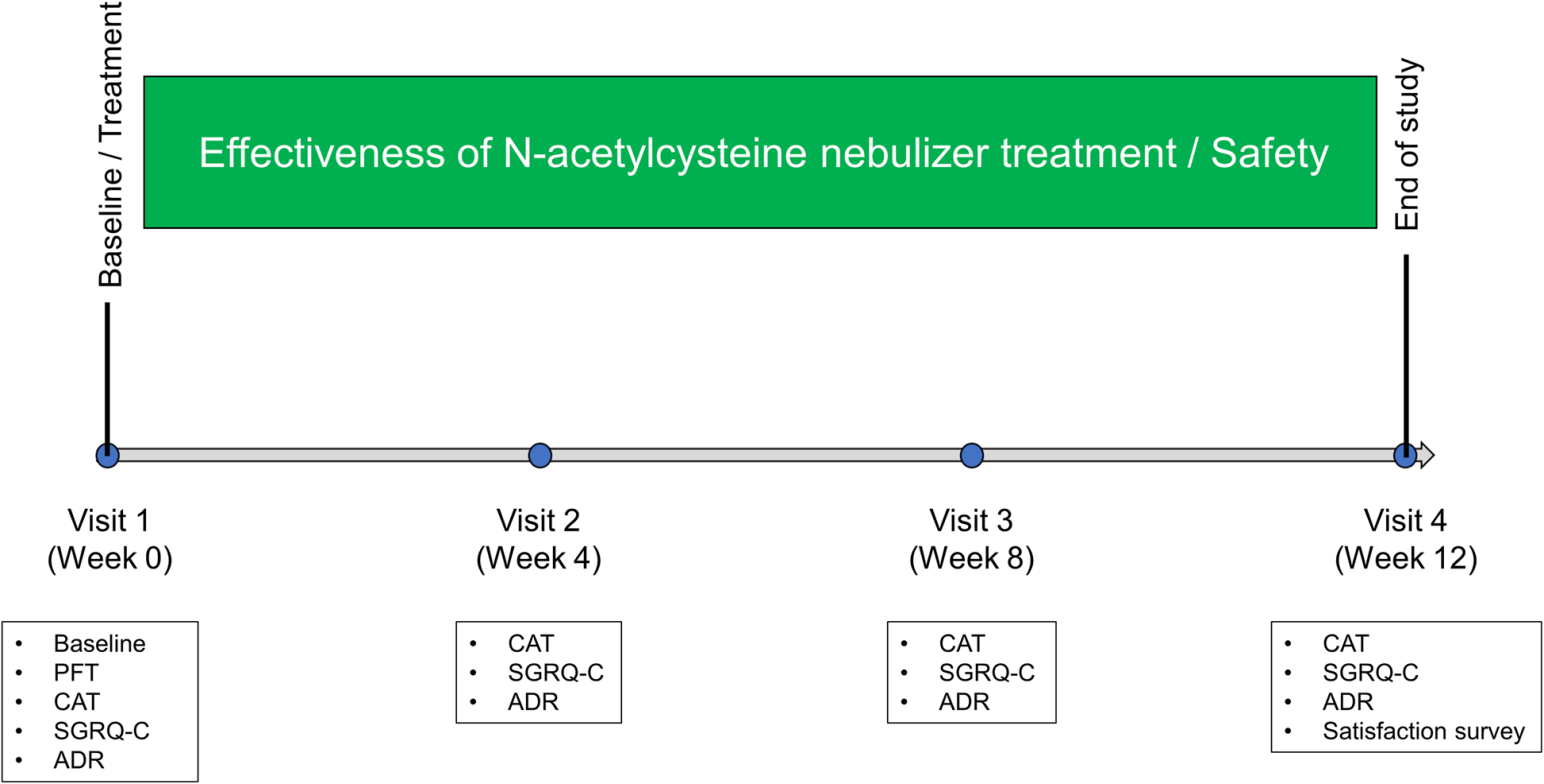
Study design. PFT = pulmonary function test, CAT = COPD assessment test, SGRQ-C = St. George’s respiratory questionnaire for COPD patients, ADR = adverse drug reaction

This was a 12-week, prospective, single-arm, open-label, phase IV multi-center trial. This trial was registered at ClinicalTrials.gov (NCT05102305, Registration Date: 20-October-2021). Ten university affiliated hospitals participated in this study. NAC (Mucomyst®) was administered via nebulizer. Each vial (4 mL) of Mucomyst contains 0.8 g NAC, diluted with normal saline in a 1:1 ratio before nebulization. NAC nebulization was performed three times daily for 12 weeks. For nebulization, P0915EM-2 EUROneb mad by Flaem Nuova was utilized. During treatment, inhalers, and other drugs remained unchanged to minimize confounding effects. Patients were assessed at baseline, 4, 8, and 12 weeks of treatment using the COPD assessment test (CAT) and St. George’s respiratory questionnaire for COPD patients (SGRQ-C) (Fig. 1).

The primary endpoint was to determine the change in CAT phlegm score at 12 weeks compared to the baseline. Secondary endpoints included changes in CAT phlegm score at 4 and 8 weeks, total CAT score at 4, 8, and 12 weeks, and SGRQ-C at 4, 8, and 12 weeks of treatment compared to the baseline, the satisfaction score and compliance with NAC assessed at 12 weeks, and the association between the satisfaction score and changes in symptom scores.

The satisfaction with nebulized NAC was assessed using a scoring system ranging from 1 to 7, where 1 indicated “extremely dissatisfied” and 7 indicated “extremely satisfied.” Furthermore, compliance with nebulized NAC was determined by counting the remaining vials at 12 weeks. The compliance percentage was calculated using the following formula: ([total sum of prescribed vials – the number of remaining vials at 12 weeks] / total sum of prescribed vials) × 100. The occurrence of adverse drug reactions (ADRs) and serious ADRs and the withdrawal rate due to ADRs was recorded at each visit.

### Inclusion and exclusion criteria

We enrolled patients aged ≥ 40 years with post bronchodilator (BD) forced expiratory volume in one second/forced vital capacity (FEV_1_/FVC) < 0.7 and CAT phlegm score ≥ 2; the patients were current or ex-smokers with smoking pack-years ≥ 10. Participants who were pregnant or breastfeeding or treated with nebulized NAC, newly prescribed mucoactive drugs, or subjected to changes in mucoactive drugs dosage within 4 weeks of treatment, and those with a history of hypersensitivity to NAC or contraindications for NAC, were excluded.

### Statistical analysis

Continuous variables are expressed as means ± standard deviations (SDs); categorical variables are expressed as numbers and percentages. Wilcoxon signed-rank test or the paired t-test was used to analyze changes from the baseline to the endpoints. Statistical analyses were performed using SPSS software (ver. 18.0; IBM Corp., Armonk, NY, USA). *P*-values < 0.05 were considered statistically significant.

### Ethics

The Institutional Review Board of each participant hospital, including Seoul St. Mary’s Hospital (approval No. KC21OSDI0637), approved the study protocols. Informed consent was obtained from all patients.

## Results

### Baseline characteristics

We evaluated the safety and efficacy of nebulized NAC in 100 enrolled patients. Safety analyses involved 99 patients (99%) who received nebulized NAC at least once and were monitored for safety-related data; one patient was excluded due to a loss of follow-up. Of the enrolled patients, their CAT phlegm score was measured at baseline and during follow-up in 91 (91%). Nine patients (9.00%) were excluded from the efficacy analysis because their CAT phlegm scores were not measured. Table 1 presents the baseline characteristics of patients included in efficacy analysis. Mean age was 71.42 ± 8.20 and 80.22% were ex-smoker and 19.78% were current. Mean smoking pack years was 40.32 ± 35.18. Mean FVC, FEV_1_, and FEV_1_/FVC were 3.94L (75.44%), 2.22L (58.50%), and 0.53.

**Table 1:** Baseline characteristics (*n* = 91)

Characteristics	Mean ± SD or number (%)
Age	71.42 ± 8.20
Male	89 (97.80%)
BMI	23.13 ± 3.14
Smoking status	
Current	18 (19.78%)
Ex	73 (80.22%)
Pack years	40.32 ± 35.18
Duration of COPD (yr)	5.69 ± 5.27
FVC (L)	3.94 ± 8.20
FVC (%)	75.44 ± 18.00
FEV_1_ (L)	2.22 ± 5.62
FEV_1_ (%)	58.50 ± 19.43
FEV_1_/FVC	0.53 ± 0.13
CAT phlegm	3.47 ± 1.06
CAT total	18.68 ± 7.55
SGRQ-C	43.05 ± 22.23

*SD* standard deviation, *BMI* body mass index, *COPD* chronic obstructive pulmonary disease, *yr* year, *FVC* forced vital capacity, *FEV*_*1*_ forced expiratory volume in one second, *CAT* COPD assessment test, *SGRQ-C* St. George’s respiratory questionnaire for COPD patients

### Efficacy of nebulized NAC

#### Primary endpoint

**Fig. 2:**
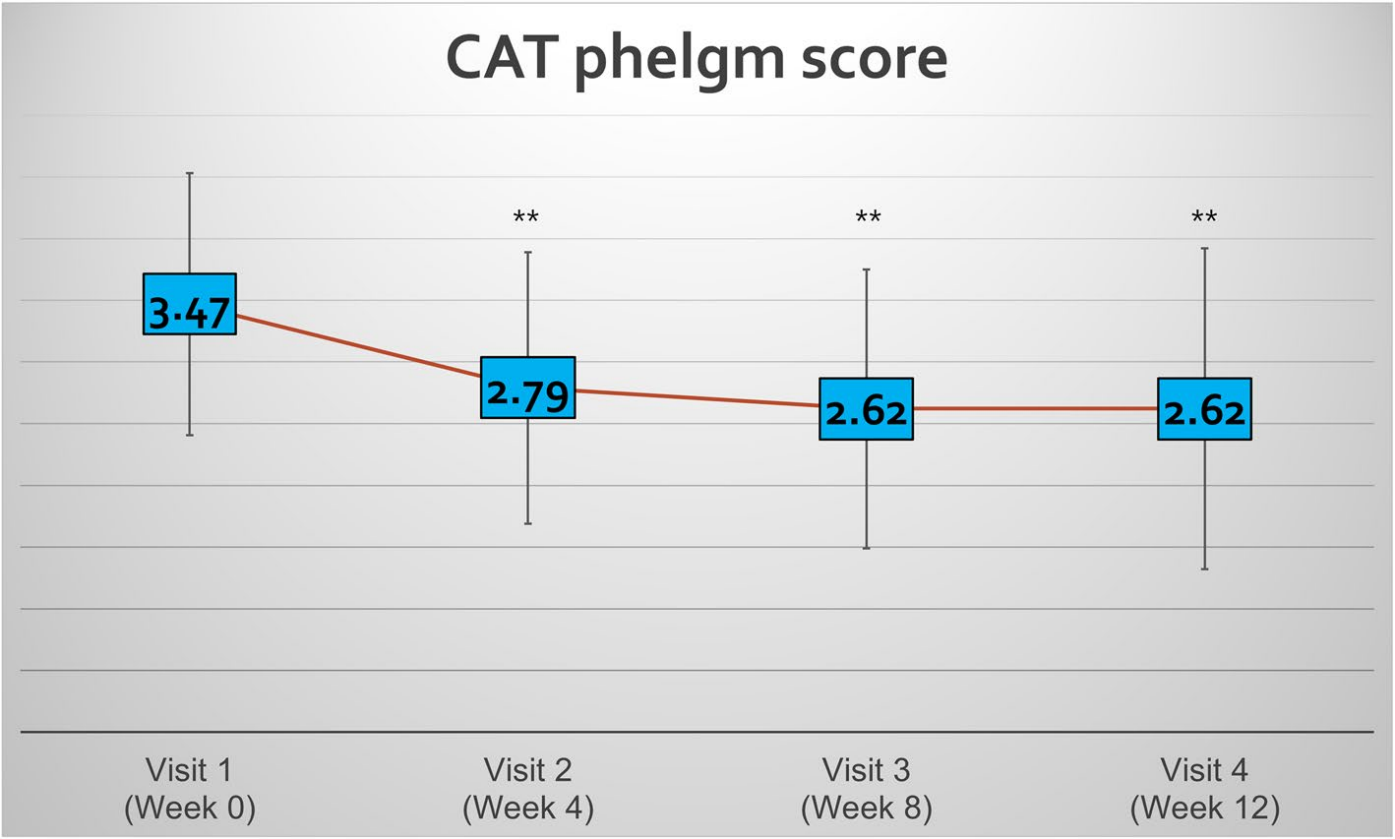
Change in CAT score after 12 weeks of nebulized NAC (*n* = 91). ^**^p < 0.01. CAT = COPD assessment test, NAC = N-acetylcysteine

The primary endpoint was the change in CAT phlegm score at 12 weeks compared to the baseline. The CAT phlegm score at baseline was 3.47 ± 1.06, whereas after 12 weeks of nebulized NAC score significantly decreased to 2.62 ± 1.30 (*p* < 0.01; Fig. 2). The primary endpoint was met.

#### Secondary endpoint

The secondary endpoints were the CAT phlegm score at 4 and 8 weeks of treatment. There were significant reductions in the score at 4 and 8 weeks of treatment (2.79 ± 1.10, *p* < 0.01; 2.62 ± 1.13, *p* < 0.01, respectively). Furthermore, the CAT total and cough scores were significantly lower after 12 weeks of treatment compared to the baseline (Table 2). Notably, significant reductions were observed in CAT total and energy score at 4 weeks and CAT total, cough, and dyspnea scores at 8 weeks of treatment.

**Table 2:** Changes in CAT score by 12 weeks NAC treatment (*n* = 91)

	Component	Baseline	4 weeks	*p* value	8 weeks	*p* value	12 weeks	*p* value
CAT	Total	18.68	16.51	0.02	16.47	< 0.00	16.63	0.02
CAT 1	Cough	2.37	2.08	0.11	1.87	< 0.01	1.87	< 0.01
CAT 2	Phlegm	3.47	2.79	< 0.01	2.62	< 0.01	2.62	< 0.01
CAT 3	Chest tightness	1.93	1.79	0.82	1.85	0.89	1.78	0.50
CAT 4	Dyspnea	3.70	3.51	0.13	3.42	0.01	3.53	0.18
CAT 5	Activity	1.24	1.14	0.67	1.38	0.21	1.47	0.10
CAT 6	Confident	1.60	1.50	0.91	1.58	0.92	1.51	0.57
CAT 7	Sleep	1.78	1.62	0.38	1.53	0.13	1.57	0.25
CAT 8	Energy	2.64	2.23	0.04	2.40	0.18	2.56	0.64

*CAT* COPD assessment test

The mean SGRQ-C phlegm scores were 89.41 ± 20.71 at baseline and 81.12 ± 22.40, 78.56 ± 22.40, and 78.17 ± 22.89 at 4, 8, and 12 weeks, respectively. The mean change in sputum score from baseline was ˗ 8.08, ˗ 10.23, and ˗ 11.13 points at 4, 8, and 12 weeks, respectively (*p* < 0.01). However, no significant differences were observed in the SGRQ-C total score.

Table 3 presents the patients’ satisfaction regarding the effectiveness of NAC at 12 weeks of treatment. Of the 89 respondents, most (53.85%) expressed satisfaction with the treatment efficacy. Table 4 exhibits the compliance of NAC during the 12-week study period. The mean compliance with NAC was 79.03 ± 25.58%; approximately 62.22% of the patients had a compliance ≥ 80%. Although a significant correlation was observed between compliance and satisfaction score (*r* = 0.43, *p* < 0.01), no associations were observed between the improvement of symptoms and compliance.

**Table 3:** Patients’ satisfaction regarding the effect of nebulized NAC (*n* = 89)

Satisfaction	Number (%)
Extremely dissatisfied	2 (2.25%)
Very dissatisfied	4 (4.49%)
Somewhat dissatisfied	14 (15.73%)
Neither satisfied nor dissatisfied	20 (22.47%)
Somewhat satisfied	34 (38.20%)
Very satisfied	13 (14.61%)
Extremely satisfied	2 (2.25%)

*NAC* N-acetylcysteine

**Table 4:** The compliance of nebulized NAC (*n* = 90)

Group	Number (%)
Compliance < 50%	11 (12.22%)
50% ≤ Compliance < 60%	4 (4.44%)
60% ≤ Compliance < 70%	9 (10.00%)
70% ≤ Compliance < 80%	10 (11.11%)
80% ≤ Compliance < 90%	10 (11.11%)
90% ≤ Compliance	46 (51.11%)

*NAC* N-acetylcysteine

#### ADR

The incidence of ADR in the safety evaluation analysis group was 8.08% (8/99 patients), whereas chest discomfort was reported in 4.04% (4/99) of the patients. Furthermore, chest pain, pyrexia, swelling face, nausea, decreased appetite, dizziness, and dyspnea were observed in 1.01% (1/99) of the patients (Table 5). Notably, no serious ADRs were reported.

**Table 5:** Adverse events of nebulized NAC (*n* = 99)

Type of adverse events	Number of cases (%)	Total count
General disorders and administration site conditions	6 (6.06)	7
Chest discomfort	4 (4.04)	4
Chest pain	1 (1.01)	1
Pyrexia	1 (1.01)	1
Swelling face	1 (1.01)	1
Gastrointestinal disorders	1 (1.01)	1
Nausea	1 (1.01)	1
Metabolism and nutrition disorders	1 (1.01)	1
Decreased appetite	1 (1.01)	1
Nervous system disorders	1 (1.01)	1
Dizziness	1 (1.01)	1
Respiratory, thoracic, and mediastinal disorders	1 (1.01)	1
Dyspnea	1 (1.01)	1

*NAC* N-acetylcysteine

## Discussion

Phlegm and cough are prevalent symptoms in COPD patients and can significantly impact quality of life. Chronic bronchitis (CB) is characterized by persistent phlegm and cough, with prevalence ranging from 14–74% [10]; it is associated with a higher symptom burden and increased risk for exacerbations [11]. Therefore, effective management of these symptoms and promoting sputum expectoration are crucial in COPD patient care.

Various approaches have been investigated to enhance sputum clearance. Oscillatory positive expiratory pressure (OPEP) devices are intended to facilitate sputum clearance and reduce cough. The use of OPEP devices has demonstrated improvements in phlegm symptoms [12]. However, the evidence supporting its use remains limited and this device is not yet widely utilized in clinical practice. Furthermore, hypertonic saline may be beneficial for promoting sputum expectoration. However, a recent study reported that it failed to significantly improve lung function or patient-reported outcomes [13].

The most widely used and convenient method of promoting sputum expectoration is the administration of mucoactive agents. Among these agents, NAC is a common drug that controls phlegm symptoms in CB and COPD patients. Previous studies have revealed the beneficial effects of NAC; a meta-analysis demonstrated its significant role in reducing exacerbations [14]. However, most evidence supporting NAC effectiveness has come from studies that have used high doses (1200 mg/day). Unfortunately, high-dose NAC may not be readily available worldwide. Furthermore, despite the use of NAC, some patients may still experience persistent phlegm symptoms. Therefore, there remains an unmet need for additional approaches to alleviate sputum symptoms in COPD patients.

Few studies have evaluated the impact of nebulized NAC on sputum. Hirsch et al*.* [15] demonstrated that nebulized NAC, administered at 10–20% concentrations, can significantly reduce sputum consistency. Similarly, Gallon et al*.* [16] conducted a study involving 10 thoracotomy patients and observed that nebulized NAC effectively reduces sputum viscosity and facilitates expectoration while increasing the weight of expectorated sputum. Consistent with these studies, we confirm the effectiveness of nebulized NAC in reducing phlegm symptoms.

The CAT is a simple and convenient scoring system used to assess the quality of life in COPD patients. Of the 8 items in the CAT score, the scores for cough and phlegm are associated with CB. Previous studies have defined CB as the CAT scores for cough and phlegm ≥ 3; this definition of CB has been predictive of more severe symptoms and high-risk patient groups [11]. In addition, CAT scores for cough and phlegm demonstrate a significant association with bronchial wall thickening observed in computed tomography [17]. Higher CAT scores for cough and phlegm are associated with more severe COPD [18]. The key advantage of the CAT score is its ease of use in routine clinical practice. Therefore, the CAT phlegm score is a valuable tool to evaluate the effects of mucoactive drugs on COPD patients. Evaluating the dominant characteristics of patients can provide valuable insights for tailoring treatment approaches [5].

Our study has several strengths. First, it is the first long-term study to establish the effectiveness of nebulized NAC. Hirsch et al*.* [15] evaluated sputum consistency before and after 30 min of nebulization, and Gallon et al*.* [16] assessed the effects of NAC over a brief period of 2 days; however, we evaluated the effects of nebulized NAC over an extended period of 12 weeks. Second, our study had a larger sample size and a more homogenous population compared to previous studies. Although Hirsch et al*.* [15] enrolled 70 COPD patients, they did not particularly focus on phlegm symptoms. Similarly, Gallon et al*.* [16] enrolled only 10 heterogenous COPD patients. Third, our study was a multicenter prospective trial, which enhances the quality of evidence obtained compared to previous studies. We evaluated the practicality of nebulized NAC in outpatient settings, with highly applicable results to clinical practice. We have established the effectiveness of nebulized NAC over 12 weeks; notably, patient compliance with the treatment regimen exceeded our expectations. Fourth, the chronic use of inhaled corticosteroids is associated with potentially adverse effects, including pneumonia, tuberculosis, and bone fractures [19–21]. However, NAC is safe and free from adverse effects associated with corticosteroids. COPD is a chronic disease, and long-term treatment is necessary. In this study, we have, for the first time, proven the long-term safety of nebulized NAC in patients with COPD.

Exposure to cigarette smoke contributes to bronchial inflammation and oxidative stress, which are significant factors in the pathophysiology of COPD. NAC, a compound known for its antioxidant properties, is thought to decrease oxidative stress in lung tissue by restoring glutathione levels. Therefore, NAC treatment could modify the oxidant/antioxidant imbalance in the lungs, potentially reducing respiratory symptoms, exacerbations, and the deterioration of lung function in COPD patients. However, literature data are controversial. Schermer and colleagues aimed to investigate whether treatment with inhaled corticosteroid (fluticasone propionate) or oral NAC are effective in primary care patients. Unfortunately, neither fluticasone propionate nor oral NAC decreased exacerbation [22]. Conversely, in large controlled clinical trials involving patients with moderate to severe stable COPD, the combination of fluticasone furoate/vilanterol enhanced lung function, reduced respiratory symptoms, and decreased the number of COPD exacerbations, including hospitalizations related to COPD [23]. Probably, in COPD, oral antioxidants or inhaled corticosteroids alone are not sufficient to reduce oxidative stress and inflammation. However, in our study, we demonstrated that inhaled NAC significantly decreased symptoms of chronic bronchitis. Thus, we may assume that inhaled forms of antioxidants can be more effective in COPD. However, further study is needed to prove this hypothesis.

However, this study also has several limitations. First, although it was a prospective multicenter trial, it was still a single-arm study without a placebo. Therefore, our results should be interpreted with caution. Further randomized, double-blind prospective trials are necessary to strengthen the evidence. Second, we did not measure sputum consistency or volume, which are essential factors for validating the effect of sputum expectoration. Third, the primary outcome tool used in this study, the CAT phlegm score, is not widely used. Limited studies have designated the CAT phlegm score as the primary endpoint [24]. Fourth, NAC nebulization three times daily for 12 weeks may not be easy in real clinical practice. Lastly, it has been shown that inhaled NAC can increase bronchial hyperresponsiveness in patients with asthma. Although exclusion criteria included history of hypersensitivity reactions to NAC, we did not specifically exclude patients with asthma. However, we tried to enroll pure COPD patients (age ≥ 40, post BD FEV_1_/FVC < 0.7, and current or ex-smokers with ≥ 10 pack-years smoking) in this study.

## Conclusions

In this study, we have established the effectiveness and safety of nebulized NAC over 12 weeks. Furthermore, the treatment was well-tolerated, with good overall compliance; no serious ADRs were reported. Importantly, more than half of the participants expressed satisfaction with the effects of nebulized NAC therapy.

## Data Availability

The datasets used and/or analyzed during the current study are available from the corresponding author on reasonable request.
